# P-1392. Sexual History Documentation Among Veterans with a Sexually Transmitted Infection (STI)

**DOI:** 10.1093/ofid/ofae631.1568

**Published:** 2025-01-29

**Authors:** Morgan C Johnson, Amy Ratliff, Mary S Dietrich, Todd Hulgan, Linda Chia, Deonni P Stolldorf, Robert S Dittus, Carol Callaway-Lane, Christianne Roumie

**Affiliations:** Tennessee Valley Healthcare System, Mount Juliet, Tennessee; Tennessee Valley Healthcare System, Nashville, Department of Veterans Affairs, Nashville, Tennessee; Vanderbilt University, Nashville, Tennessee; Tennessee Valley Healthcare System and Vanderbilt University Medical Center, Nashville, Tennessee; VA, Bellevue, Washington; Vanderbilt University, Nashville, Tennessee; Vanderbilt University Medical Center, Nashville, Tennessee; Tennessee Valley Healthcare System, Mount Juliet, Tennessee; VA Tennessee Valley Healthcare System, Nashville, Tennessee

## Abstract

**Background:**

Sexual health discussions in routine healthcare encounters can help identify sexually transmitted infection (STI) risk. We measured the prevalence of sexual history completion in Veterans with an STI and association between STI testing and offer of HIV pre-exposure prophylaxis (PrEP).

**Table 1.**
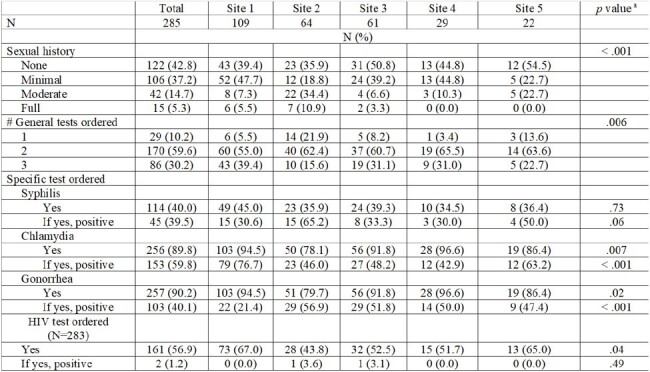
Summaries of levels of sexual history and STI tests ordered by sample sites (N=285) a Pearson Chi-Square Test of Independence

**Methods:**

We conducted a retrospective review of a Veteran cohort with a STI documented on a clinical encounter between December 2020-December 2021 at five Veterans Affairs (VA) facilities in the southern United States. Primary care, emergency room, and specialty clinic visits were included, and infectious disease clinic visits excluded. STI was defined as a positive result for chlamydia, gonorrhea, and/or syphilis. The CDC’s definition of an appropriate sexual history utilizing the “5 Ps” (partners, practices, protection, pregnancy, and past STIs) was used as the reference standard. Level of sexual history taken was categorized as none (0 Ps), minimal (1-2 Ps), moderate (3 Ps), or full (4-5 Ps for a male, 5 Ps for female). Chi-square tests and logistic regression were used for analyses.

**Table 2.**
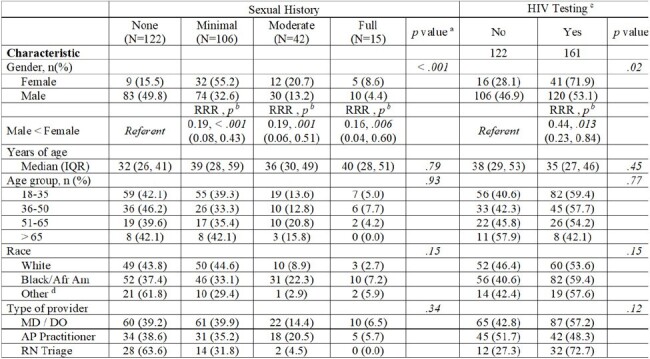
Associations of demographic characteristics with extent of sexual history taken during the encounter and HIV testing controlling for VA site. (N=285) a Multinomial logistic regression, log likelihood test b p-value of relative risk ratio (RRR), adjusted for study site c Number of sites included = 283 d Other included American-Indian/Alaska Native (1), Asian(1), Hispanic/Latino(6), Native Hawaiian/Pacific Islander(2), Multiple races(6)

**Results:**

We included 285 records in the review. A sexual history was not conducted (0 Ps) in 43% (122/285) of encounters. There were statistically significant differences in obtaining sexual history and number of STI tests ordered across VA facilities (p< .001 and .006, respectively). No evidence of documentation occurred for sexual practices (93%), history of STIs (78%), and sexual partners (67%). Males were less likely to receive a full sexual health history (p=.01) and HIV testing (53 vs 72%, p=.007) compared to females. Only 4 (1%) Veterans were offered PrEP. After controlling for facility, a statistically significant association between the extent of documented sexual history and STI tests ordered (p=.019). No significant associations were observed between extent of history or HIV testing for Veterans’ age, race, or provider type.

**Table 3.**
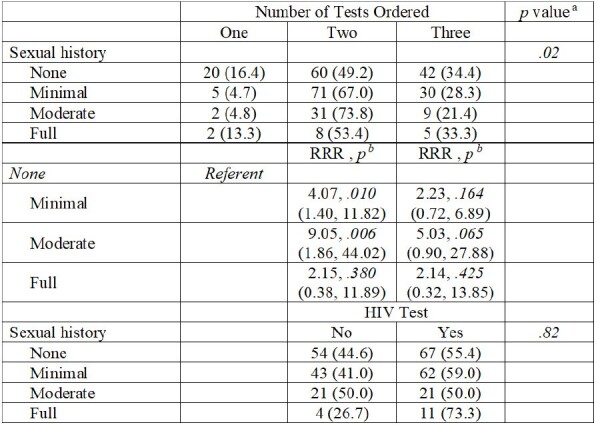
Associations of extent of sexual health history taken and tests ordered after controlling for VA site. (N=285) a Multinomial logistic regression, log likelihood test b p-value of relative risk ratio (RRR), adjusted for study site c Other included American-Indian /Alaska Native1), Asian (1), Hispanic/Latino (6), Native Hawaiian/Pacific Islander (2), Multiple races (6)

**Conclusion:**

Incomplete assessment of sexual history was common in outpatient visits and varied by facility for Veterans with a STI. There was a relationship between increasing level of sexual history and number of STI tests ordered. Future work includes identifying systematic interventions to support standardization of sexual history taking.

**Disclosures:**

**All Authors**: No reported disclosures

